# Autosomal recessive congenital cataract in consanguineous Pakistani families is associated with mutations in *GALK1*

**Published:** 2010-04-15

**Authors:** Afshan Yasmeen, S. Amer Riazuddin, Haiba Kaul, Sadia Mohsin, Mohsin Khan, Zaheeruddin A. Qazi, Idrees A. Nasir, Ahmad U. Zafar, Shaheen N. Khan, Tayyab Husnain, Javed Akram, J. Fielding Hejtmancik, Sheikh Riazuddin

**Affiliations:** 1 National Centre of Excellence in Molecular Biology, University of the Punjab, Lahore, Pakistan; 2 Layton Rahmatulla Benevolent Trust, Lahore, Pakistan; 3 Allama Iqbal Medical College, University of Health Sciences, Lahore, Pakistan; 4 Ophthalmic Genetics and Visual Function Branch, National Eye Institute, National Institutes of Health, Bethesda, MD

## Abstract

**Purpose:**

To identify the pathogenic mutations responsible for autosomal recessive congenital cataracts in consanguineous Pakistani families.

**Methods:**

All affected individuals underwent detailed ophthalmologic and medical examination. Blood samples were collected and genomic DNA was extracted. A genome-wide scan was performed with polymorphic microsatellite markers on genomic DNA from affected and unaffected family members and logarithm of odds (LOD) scores were calculated. All coding exons of galactokinase (*GALK1*) were sequenced to identify pathogenic lesions.

**Results:**

Clinical records and ophthalmological examinations suggested that affected individuals have nuclear cataracts. Linkage analysis localized the critical interval to chromosome 17q with a maximum LOD score of 5.54 at θ=0, with D17S785 in family PKCC030. Sequencing of *GALK1*, a gene present in the critical interval, identified a single base pair deletion: c.410delG, which results in a frame shift leading to a premature termination of GALK1: p.G137fsX27. Additionally, we identified a missense mutation: c.416T>C, in family PKCC055 that results in substitution of a leucine residue at position 139 with a proline residue: p.L139P, and is predicted to be deleterious to the native GALK1 structure.

**Conclusions:**

Here, we report pathogenic mutations in *GALK1* that are responsible for autosomal recessive congenital cataracts in consanguineous Pakistani families.

## Introduction

Congenital cataracts are one of the major causes of vision loss in children worldwide [1,2]. It can occur in an isolated fashion or as one component of a syndrome affecting multiple tissues [3]. Congenital cataracts vary markedly in severity and morphology, affecting the nuclear, cortical, polar, or subcapsular parts of the lens, or in severe cases the entire lens [4]. To-date, fourteen loci have been associated with autosomal recessive cataracts with seven of these also causing autosomal dominant cataracts [5-18]. Of these loci, mutations in nine genes, eph-receptor type-A2 (*EPHA2*), connexin50 (*GJA8*), glucosaminyl (N-acetyl) transferase 2 (*GCNT2*), heat-shock transcription factor 4 (*HSF4*), lens intrinsic membrane protein (*LIM2*), beaded filament structural protein 1 (*BFSP1*), alphaA-crystallin (*CRYαA*), betaB1-crystallin (*CRYβB1*), and betaB3-crystallin (*CRYβB3*) have been found [5,7,9,12,14-18].

Galactokinase (*GALK1*) is involved in the first step of metabolism of galactose, the conversion of galactose to galactose-1-phosphate at the expense of ATP. In the absence of GALK1 the accumulating galactose is converted to galactitol by aldose reductase. Human *GALK1* has been mapped to chromosome 17q25.1; it contains 8 exons and encodes for a 392 amino acid protein harboring two ATP binding sites [19]. Stambolian and colleagues first identified mutations in *GALK1* in families with cataracts [19].

Here, we report two consanguineous Pakistani families with congenital cataracts. The critical interval was localized to chromosome 17q with significant two-point logarithm of odds (LOD) scores for both families and haplotype analyses supported the linkage results. Sequencing of *GALK1* identified a single base pair deletion that results in premature termination and a missense mutation that leads to a non-conservative substitution in these two families, respectively. These variations were not present in 96 ethnically matched control samples.

## Methods

### Clinical ascertainment

A total of 100 consanguineous Pakistani families with nonsyndromic cataracts were recruited to participate in a collaborative study between the National Centre of Excellence in Molecular Biology, Lahore, Pakistan, and the National Eye Institute, Bethesda, MD, to identify novel loci associated with congenital cataracts. Institutional Review Board (IRB) approval was obtained from the National Centre of Excellence in Molecular Biology and the National Eye Institute. The participating subjects gave informed consent consistent with the tenets of the Declaration of Helsinki. A detailed medical history was obtained by interviewing family members. Ophthalmic examinations were conducted with slit-lamp microscopy. Approximately 10 ml of blood samples were drawn from affected and unaffected members of the family and stored in 50 ml Sterilin® falcon tubes (BD Biosciences, San Jose, CA) containing 400 μl of 0.5 M EDTA. Blood samples were kept at −20 °C for long- term storage.

### DNA extraction

DNA was extracted by a nonorganic method as described previously [5,10]. Briefly, aliquots of 10 ml blood samples were mixed with 35 ml of TE buffer (10 mM Tris-HCl, 2 mM EDTA, pH 8.0) and the TE-blood mixture was centrifuged at 3,000 rpm (1,800× g) for 20 min. The supernatant was discarded and the pellet was re-suspended in 35 ml of TE buffer and centrifuged at 3,000 rpm (1,800× g) for 20 min. The TE washing was repeated for 2–3 times and the washed pellet was re-suspended in 2 ml of TE. 6.25 ml of protein digestion cocktail (50 μl [10 mg/ml] of proteinase K, 6 ml TNE buffer [10 mM Tris HCl, 2 mM EDTA, 400 mM NaCl] and 200 μl of 10% sodium dodecyl sulfate) was added to the re-suspended pellets and incubated overnight in a shaker (250 rpm) at 37 °C. The digested proteins were precipitated by adding 1 ml of 5 M NaCl, followed by vigorous shaking and chilling on ice for 15 min. The precipitated proteins were pelleted by centrifugation at 3,000 rpm (1,800× g) for 20 min and removed. The supernatant was mixed with equal volumes of phenol/chloroform/isoamyl alcohol (25:24:1) and the aqueous layer containing the genomic DNA was carefully collected. The DNA was precipitated with isopropanol and pelleted by centrifugation at 4,000 rpm (2,400× g) for 15 min. The DNA pellets were washed with 70% ethanol and dissolved in TE buffer. The DNA concentration was determined with a SmartSpec plus Bio-Rad Spectrophotometer (Bio-Rad, Hercules, CA).

### Genotype analysis

A genome-wide scan was performed with 382 highly polymorphic fluorescent markers from the ABI PRISM Linkage Mapping Set MD-10 (Applied Biosystems, Foster City, CA) having an average spacing of 10 cM. Multiplex polymerase chain reaction (PCR) was completed in a GeneAmp PCR System 9700 thermocycler (Applied Biosystems). Briefly, each reaction was performed in a 5 μl mixture containing 40 ng genomic DNA, various combinations of 10 mM dye-labeled primer pairs, 0.5 ml 10× GeneAmp PCR Buffer (Applied Biosystems), 1 mM dNTP mix, 2.5 mM MgCl_2_, and 0.2 U *Taq* DNA polymerase (Applied Biosystems). Initial denaturation was performed for 5 min at 95 °C, followed by 10 cycles of 15 s at 94 °C, 15 s at 55 °C, and 30 s at 72 °C and then 20 cycles of 15 s at 89 °C, 15 s at 55 °C, and 30 s at 72 °C. The final extension was performed for 10 min at 72 °C. PCR products from each DNA sample were pooled and mixed with a loading cocktail containing HD-400 size standards (Applied Biosystems). The resulting PCR products were separated in an ABI 3100 DNA Analyzer (Applied Biosystems) and genotypes were assigned with GeneMapper software (Applied Biosystems).

### Linkage analysis

Two-point linkage analyses were performed using the FASTLINK version of MLINK from the LINKAGE Program Package (provided in the public domain by the Human Genome Mapping Project Resources Centre, Cambridge, UK) [20,21]. Maximum LOD scores were calculated with ILINK from the LINKAGE Program Package. Autosomal recessive cataract was analyzed as a fully penetrant trait with an affected allele frequency of 0.001. The marker order and distances between the markers were obtained from the Marshfield database and the National Center for Biotechnology Information (NCBI) chromosome 17 sequence maps. For the initial genome scan, equal allele frequencies were assumed, while for fine mapping allele frequencies were estimated from 96 unrelated and unaffected individuals from the Punjab province of Pakistan.

### Mutation screening

Primer pairs for individual exons were designed using the primer3 program. The sequences and annealing temperatures are given in Table 1. Amplifications were performed in a 25 μl reaction containing 50 ng of genomic DNA, 400 nM of each primer, 250 μM of dNTPs, 2.5 mM MgCl_2_ and 0.2 U *Taq* DNA polymerase in the standard PCR buffer provided by the manufacturer (Applied Biosystems). PCR amplification consisted of a denaturation step at 96 °C for 5 min followed by 40 cycles, each consisting of 96 °C for 30 s followed by 57 °C (or primer set-specific annealing temperature; see Table 1) for 30 s and 72 °C for 1 min. PCR products were analyzed on a 2% agarose gel and purified by ethanol precipitation. The PCR primers for each exon were used for bidirectional sequencing using BigDye Terminator Ready reaction mix (Applied Biosystems), according to the manufacturer’s instructions. Sequencing products were precipitated and resuspended in 10 μl of formamide (Applied Biosystems) and denatured at 95 °C for 5 min. Sequencing was performed in an ABI PRISM 3100 Automated Sequencer (Applied Biosystems) and sequencing results were assembled with ABI PRISM sequencing analysis software version 3.7 and analyzed with SeqScape software (Applied Biosystems).

**Table 1:** Primer sequences and annealing temperatures of *GALK1*.

**Exon**	**Forward**	**Reverse**	**Product size (bp)**	**Annealing temperature (°C)**
1	GAACCGGCTGAGGTCTGGG	CTTCCTCCCTTCCAACGTGGG	496	55
2	AGCTGGCCCTCAGGATCTTC	GGGCATCAGTTTCCTCATCTG	488	55
3	GTTGGTGGCTTCTGACAATTG	GTGGCTTCAATGACACTCCAG	384	57
4	CAGGTGGTCCCAGCTTCTAC	ACCTGGGGTGGAGTTACAATG	387	55
5	CCCTGCACTCAGCAGCTC	AAGCAGCCCTGCTGAGATTG	449	55
6	CCACCCTTCACCGTCCAGC	CCATAAACCCCAGGCACAGC	413	57
7	GTGGCGACTACAGAGCCTTTG	CGGCTGCTTGAGAGAGGTAG	422	55
8	CTGCACGGTGACACTGCTG	TTGAGCACCCGGATATGGAAG	307	55

### Prediction analysis

Evolutionary conservation of the mutated amino acids in other GALK1 orthologs was examined using the UCSC genome browser. The degree of evolutionary conservation of positions at which missense mutations exist and the possible impact of an amino acid substitution on the structure of GALK1 was examined with SIFT and PolyPhen tools available online.

## Results

**Figure 1:**
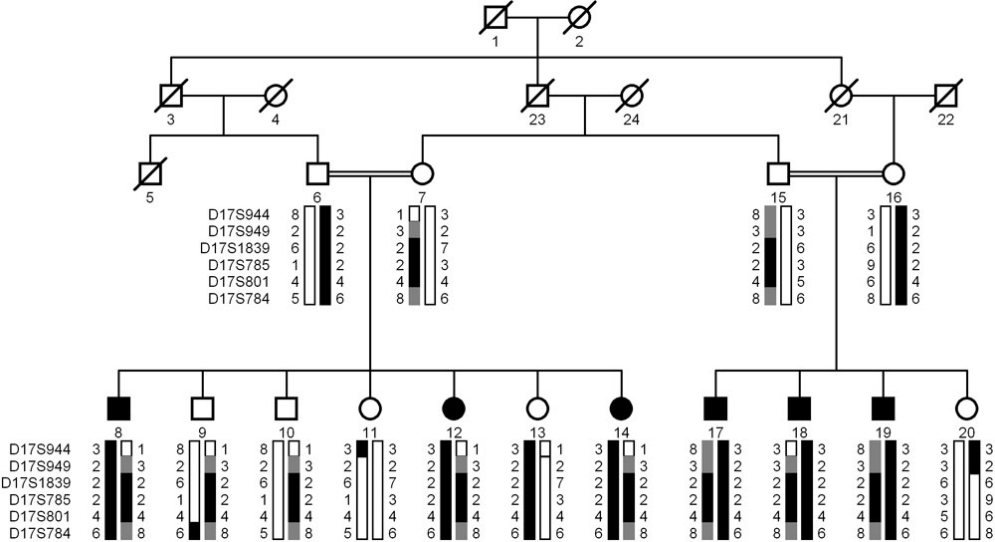
Pedigree drawing and haplotypes of chromosome 17q markers of family PKCC030. Squares are males, circles are females, and filled symbols are affected individuals; the double line between individuals indicates consanguinity and the diagonal line through a symbol is a deceased family member. The haplotypes of 6 adjacent chromosome 17q microsatellite markers are shown with alleles forming the risk haplotype are shaded black, alleles co-segregating with cataracts but not showing homozygosity are shaded gray and alleles not co-segregating with cataracts are shown in white.

**Figure 2:**
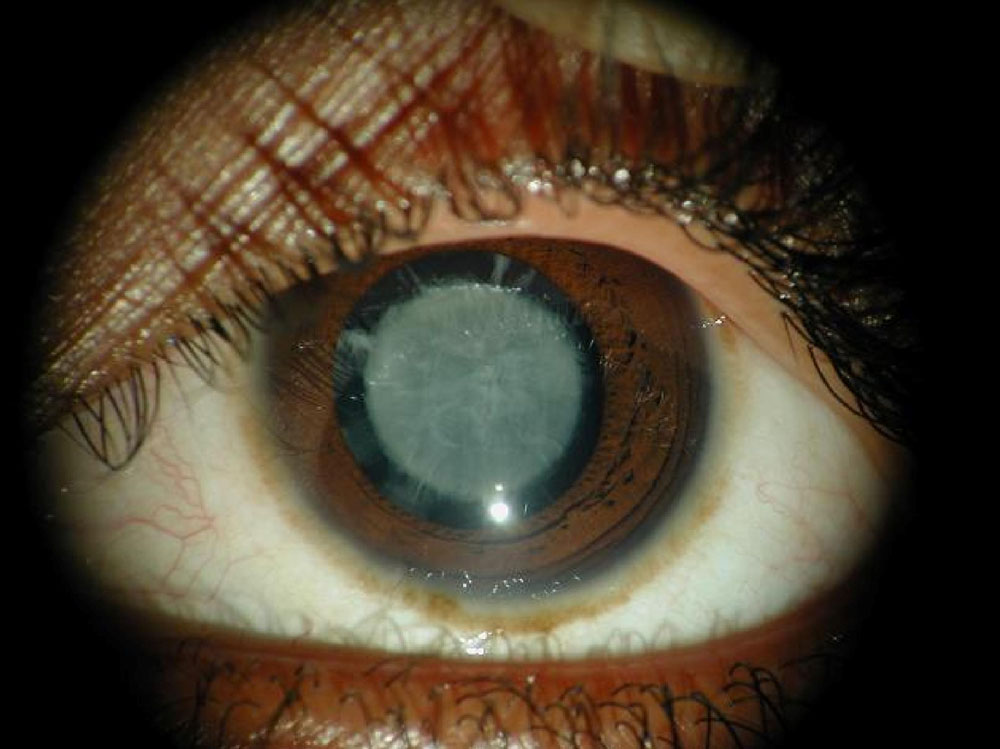
Slit lamp photographs of the affected individual 14 of family PKCC030 show nuclear cataracts that developed in early infancy.

A large consanguineous family, PKCC030 (Figure 1) consisting of six affected and nine unaffected individuals was recruited from the Punjab province of Pakistan. A detailed medical history was obtained from all family members. Medical records of previously conducted ophthalmic examinations with slit lamp biomicroscopy were suggestive of nuclear cataract in affected individuals of PKCC030 (Figure 2). According to the medical records available to us all the affected individuals developed cataract in the first year of their life except individual 14 who developed cataract in the 2nd year of her life. The medical examination concluded that affected individuals did not present any extra-ocular anomalies, although according to family elders, higher intake of dairy products usually results in vomiting and diarrhea in all affecteds.

Initially, linkage to known autosomal recessive cataract loci was excluded by haplotype analysis using closely flanking markers (data not shown). Next, we completed a genome-wide scan with 382 fluorescently-labeled short tandem repeat (STR) markers. A maximum two-point LOD score of 5.54 at θ=0 was obtained with D17S785 during the genome-wide scan (Table 2). Additional STR markers from the Marshfield database were designed to analyze the critical interval, which further provided evidence of linkage to chromosome 17q with LOD scores of 5.49 and 2.76 at θ=0 with markers D17S1839 and D17S801, respectively (Table 2).

**Table 2:** Two-point LOD scores of chromosome 17q markers.

**Marker**	**cM**	**Mb**	**0**	**0.01**	**0.05**	**0.1**	**0.2**	**0.3**	**0.4**	**Z_max_**	**θ_max_**
D17S944*	82.56	61.43	-∞	−4.07	−1.55	−0.68	−0.14	−0.03	−0.02	0.40	0.85
D17S949*	93.27	68.46	−2.50	−0.38	0.19	0.32	0.29	0.15	0.04	0.33	0.13
D17S1839	102.46	73.80	5.49	5.37	5.16	4.65	3.57	2.37	1.13	5.49	0.00
D17S785*	103.53	74.43	5.54	5.43	5.21	4.70	3.62	2.43	1.19	5.62	0.00
D17S801	103.53	74.50	2.76	2.69	2.47	2.19	1.58	0.95	0.38	2.76	0.00
D17S784	116.86	77.80	-∞	−0.88	0.24	0.49	0.45	0.25	0.08	0.52	0.13

The asterisk indicates that the STR markers were included in the genome-wide scan.

Visual inspection of the haplotypes supported the results of linkage analysis (Figure 1). This places the critical interval in a 34.3 cM (16.37 Mb) interval, flanked by markers D17S944, proximally and marker D17S784, distally. Lack of homozygosity in alleles of affected individuals 8, 12, 14, 17, 18, and 19 at D17S949, further suggests that the pathogenic mutations resides in a 23.59 cM (9.34 Mb) interval flanked by D17S949 proximally and D17S784 distally.

**Figure 3:**
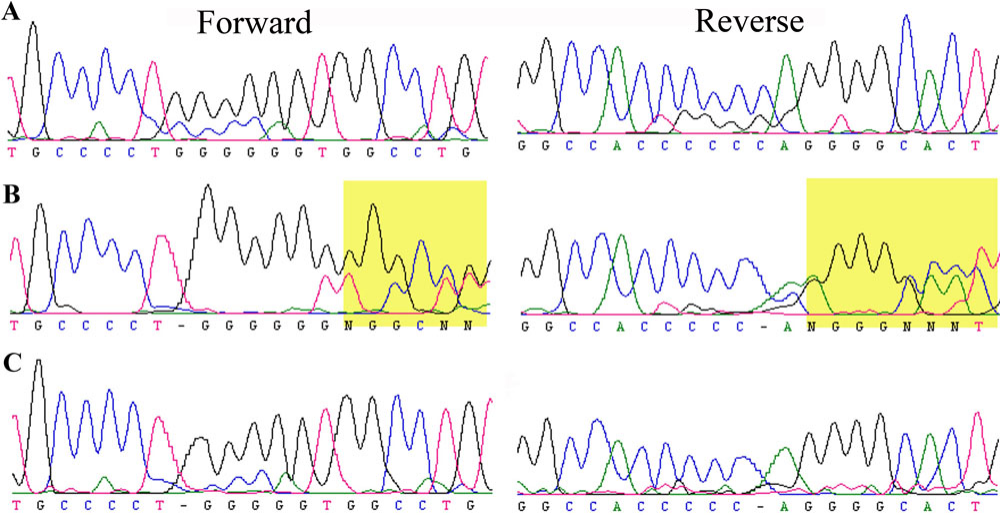
Forward and reverse sequence chromatograms illustrating a single base pair deletion in *GALK1*. **A**: Individual 11 homozygous for wild type allele. **B**: Individual 13 heterozygous for mutant and wild type allele. **C**: Individual 14 homozygous for the single base pair deletion in exon 3; c.410delG. This deletion leads to a frame shift in the open reading frame of GALK1, which results in a premature termination of the protein: p.G137fsX27.

The critical interval harbors *GALK1*, a gene previously associated with cataractogenesis [19]. We sequenced all coding exons, exon-intron boundaries and the 100 bases of the 5`- and 3`-regions of *GALK1*. We identified a homozygous single base pair deletion in exon 3: c.410delG in PKCC030 (Figure 3). This deletion leads to a frame shift in the open reading frame (ORF) of GALK1, which results in a premature termination of the protein: p.G137fsX27. This single base-pair deletion segregated with the disease phenotype in both consanguineous loops of PKCC030 and was not present in ethnically matched control samples.

**Figure 4:**
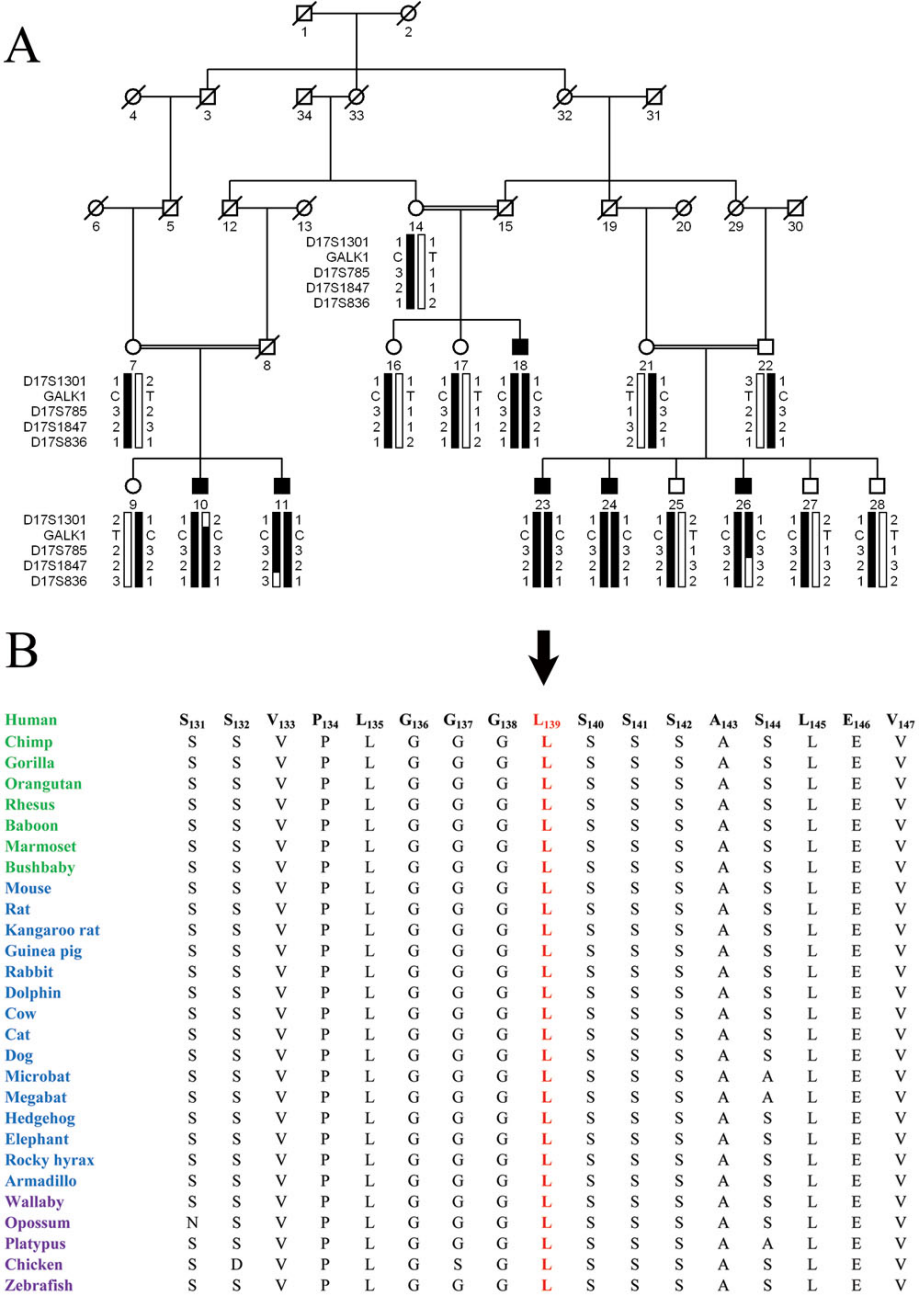
Pedigree drawing of family PKCC055 and alignment of L139 in *GALK1* orthologs. **A**: Illustration of the chromosome 17q haplotypes and segregation of c.416T>C variation with the disease phenotype. **B**: Conservation of L139 in other GALK1 orthologs is illustrated with primates colored green; placental mammals blue; and vertebrates are purple. The arrow points to amino acid L139.

Subsequent to the identification of c.410delG in PKCC030, we interrogated our entire cohort of congenital cataract families with closely spaced STR markers flanking *GALK1* to identify other families localizing to chromosome 17q. We identified one other family: PKCC055 with a two-point LOD score of 4.55 at θ=0, with marker D17S785, which was supported by haplotype analyses (Figure 4A). Bi-directional sequencing of *GALK1* identified a missense mutation: c.416T>C, which segregated with the disease phenotype in the family (Figure 4A). All affected individuals were homozygous for this mutation, whereas the unaffected individuals were heterozygous carriers of the mutation (Figure 4A). The variation was not present in 96 ethnically matched control samples.

This mutation results in substitution of the leucine residue at position 139 with a proline residue; p.L139P. The leucine residue at position 139 is highly conserved in other GALK1 orthologs as shown in Figure 4B. SIFT predictions were suggestive of the fact that the L139P substitution will affect the native three dimensional structure of GALK1 with a protein function score of 0.01 (amino acids with probabilities <0.05 are predicted to be deleterious). Likewise, position-specific independent counts (PSIC) score differences obtained from PolyPhen suggested that the L139P substitution could potentially have a deleterious effect on GALK1 structure with a PSIC score of 1.44 (a PSIC score difference >1.0 is probably damaging).

## Discussion

Here, we report pathogenic mutations in *GALK1* that are responsible for autosomal recessive congenital cataracts in two Pakistani families. Both families mapped to chromosome 17q with significant LOD scores and these results were supported by visual inspection of the haplotype analyses, localizing the critical interval to a region harboring *GALK1*. Sequencing of *GALK1* indentified a homozygous single base pair deletion in the first family, which results in premature termination of GALK1 and a missense mutation in a second family, which leads to a non-conservative substitution. These variations were not present in 96 ethnically matched control samples. This is the first report associating *GALK1* with autosomal recessive congenital cataracts in families of Pakistani origin.

Glucose is a highly consumed monosaccharide, which is converted to glucose-6-phosphate in a four step process known as the glycolytic pathway. Any disruption of these processes can potentially result in galactosemia. GALK1 is involved in the first step of metabolism of galactose, the conversion of galactose to galactose-1-phosphate at the expense of ATP. In the absence of GALK1, the accumulating galactose is converted to galactitol by aldose reductase. Accumulation and subsequent osmotic swelling of galactitol results in cataracts [22], probably due to osmotic swelling. All affected individuals reported in this study developed cataracts in their infancy whereas the unaffected heterozygous carriers of the pathogenic mutations in *GALK1* did not present any signs or symptoms of caractogenesis, even in their forties.

Identification of the pathogenic mutations in *GALK1* and the phenotype of cataracts associated with these mutations will increase our understanding of lens biology at a molecular level, which will lead to better treatments and therapeutics.
